# Characterisation of the new EpCAM-specific antibody HO-3: implications for trifunctional antibody immunotherapy of cancer

**DOI:** 10.1038/sj.bjc.6603881

**Published:** 2007-07-10

**Authors:** P Ruf, O Gires, M Jäger, K Fellinger, J Atz, H Lindhofer

**Affiliations:** 1 Department of Antibody Development, TRION Research GmbH, Martinsried, Germany; 2 Clinical Cooperation Group Molecular Oncology, GSF-Research Centre for Health, and Environment and Department of Otorhinolaryngology, Ludwig-Maximilians-University, Munich, Germany; 3 Department of Preclinical Research and Development, Fresenius Biotech GmbH, Gräfelfing, Germany; 4 CEO, TRION Pharma GmbH, Munich, Germany

**Keywords:** EpCAM (CD326), immunotherapy, trifunctional antibodies

## Abstract

Epithelial cell adhesion molecule EpCAM is a transmembrane glycoprotein that is frequently overexpressed in a variety of carcinomas. This pan-carcinoma antigen has served as the target for a plethora of immunotherapies. Innovative therapeutic approaches include the use of trifunctional antibodies (trAbs) that recruit and activate different types of immune effector cells at the tumour site. The trAb catumaxomab has dual specificity for EpCAM and CD3. In patients with malignant ascites, catumaxomab significantly increased the paracentesis-free interval, corroborating the high efficacy of this therapeutic antibody. Here, we characterised the monoclonal antibody (mAb) HO-3, that is, the EpCAM-binding arm of catumaxomab. Peptide mapping indicated that HO-3 recognises a discontinuous epitope, having three binding sites in the extracellular region of EpCAM. Studies with glycosylation-deficient mutants showed that mAb HO-3 recognised EpCAM independently of its glycosylation status. High-affinity binding was not only detected for mAb HO-3, but also for the monovalent EpCAM-binding arm of catumaxomab with an excellent *K*_D_ of 5.6 × 10^−10^ M. Furthermore, trAb catumaxomab was at least a 1000-fold more effective in eliciting the eradication of tumour cells by effector peripheral blood mononuclear cells compared with mAb HO-3. These findings suggest the great therapeutic potential of trAbs and clearly speak in favour of EpCAM-directed cancer immunotherapies.

EpCAM (CD326) is a type I transmembrane glycoprotein that acts as a Ca^2+^ independent homophilic cell adhesion molecule (Litvinov *et al*, 1994). The 39–42 kDa protein is composed of a large extracellular domain with two EGF-like repeats, a single transmembrane region, and a short cytoplasmic tail of 26 amino acids (Balzar *et al*, 1999). The high frequency of its overexpression in a multitude of human carcinomas qualifies EpCAM as a target of interest for immunotherapy. High-level EpCAM expression was demonstrated in major malignancies such as colon, stomach, and prostate cancers and in adenocarcinoma of the lung (Went *et al*, 2006). Besides being a target antigen, EpCAM is a prognostic marker in breast, ovarian, and gallbladder cancers where its overexpression correlates with poor prognosis (Gastl *et al*, 2000; Spizzo *et al*, 2002, 2006; Varga *et al*, 2004). The therapeutic potential for the inhibition of EpCAM was illustrated by a knockdown phenotype of EpCAM in carcinoma cells. Treatment of head neck, and breast cancer cells with EpCAM-specific antisense or siRNAs resulted in the reduction or even abrogation of cell proliferation, migration, and invasive capacity (Munz *et al*, 2004; Osta *et al*, 2004).

Moreover, EpCAM supports active escape from immune surveillance. Ectopic expression of EpCAM in dendritic cells blocks their ability to present MHC class II-restricted antigens (Gutzmer *et al*, 2004). All these features have encouraged the development of various EpCAM-directed anti-cancer therapies. Trifunctional antibodies (trAbs) with a dual specificity for EpCAM expressed on tumour cells and CD3 expressed on T cells represent an innovative immunotherapeutic approach to fight cancer. TrAbs induce a tri-cell complex of tumour cells, T cells, and accessory immune cells due to their unique Fc-composition of mouse IgG2a and rat IgG2b. Thereby, crosstalk between different types of redirected immune effector cells is initialised, that results in efficient killing of the tumour cells (Zeidler *et al*, 1999, 2000; Riesenberg *et al*, 2001). Encouraging first clinical results confirm those observed in *in vitro* anti-tumour potency studies. Positive indication of clinical benefit in the treatment of metastatic breast cancer came from a phase I dose escalation trial using a HER-2-specific trAb (Kiewe *et al*, 2006). Moreover, the feasibility of combining trAbs with high-dose chemotherapy for metastatic breast cancer treatment was shown in a pilot study (Stemmler *et al*, 2005). Finally, trAbs were safely applied and demonstrated convincing efficacy in patients suffering from malignant ascites (Heiss *et al*, 2005).

Here, we report on the characterisation of the new monoclonal antibody (mAb) HO-3 that constitutes the EpCAM-binding arm of the corresponding trAb catumaxomab (removab®). A detailed analysis of the binding features and anti-tumour efficacy of HO-3 in comparison with catumaxomab is presented. Furthermore, we discuss the impact of the binding kinetics with respect to immunotherapy of EpCAM-positive tumours by conventional monoclonal *vs* trifunctional antibodies.

## MATERIALS AND METHODS

### Generation of HO-3 hybridoma

Balb/c mice were immunised by intraperitoneal injection of the EpCAM-positive human colon carcinoma cell line HCT-8 (ATCC No. CCL-224). One week after a booster immunisation, the mice were killed and spleen cell preparations were fused with the mouse myeloma cell line P3X63Ag8.653 (ATCC No. CRL 1580). Supernatants of single growing clones were screened for competitive binding with anti-EpCAM mAb C215 to HCT-8 cells by flow cytometry. The isolated hybridoma clone HO-3 was further stabilised by several rounds of subcloning. It produces mouse antibodies of the subtype IgG2a.

### Antibodies and EpCAM antigen

The EpCAM-specific antibodies C215 (Bjork *et al*, 1993), VU-1D9 (Balzar *et al*, 1999), HO-3 and the anti-mouse Thy-1.2. isotype control MmT1 (Kremmer *et al*, 1989) were all produced by hybridoma technology and protein G (GE Healthcare, Uppsala, Sweden) affinity purification at TRION Research. Catumaxomab (removab®), the trAb variant of HO-3 with a dual specificity for EpCAM and CD3 and the trifunctional control antibody Bi20 (anti-CD20 × anti-CD3) were manufactured using the quadroma technology (Lindhofer *et al*, 1995) at TRION Pharma (Munich, Germany) under GMP conditions.

The extracellular domain (ECD) of the human EpCAM protein was heterologously expressed in the yeast strain *pichia pastoris* at GenYouIn Biotech (Reutlingen, Germany). C-terminal addition of a His_6_ tag allowed metal-affinity purification of the protein via Ni NTA agarose (Qiagen, Hilden, Germany).

### Establishing EpCAM glycosylation mutants

Asparagine residues within the NGT/S N-glycosylation consensus sequence at amino-acid positions 74, 111, and 198 were changed to alanines by PCR-based site directed mutagenesis. A triple knockout mutant was generated by sequential single mutations using the primers shown in Table 1. PCR amplification products were subcloned into the PCR cloning vector pDRIVE (Qiagen) before transfer into the eukaryotic expression vector pcDNA3.1-hygro^+^ (Invitrogen, Heidelberg, Germany) for transient transfection of HEK293 cells. Empty vector was used as a negative control for transfection and subsequent detection by flow cytometry. The glycosylation status of the proteins was assessed with the glycostain kit (Molecular Probes, Göttingen, Germany) according to the manufacturer's instructions.

### Construction of an EGF-like domain I EpCAM deletion mutant

Deletion of the EGF-like domain I (amino acids 27–59) was performed by overlapping PCR mutagenesis with the primers listed in Table 1. PCR fragments were subcloned into the pDRIVE vector and subsequently transferred into pcDNA3.1-hygro^+^ using the *Nhe*1 and *Xba*1 restriction enzyme sites. Transiently transfected HEK293 cells, including a positive control plasmid expressing wild-type EpCAM, were then analysed by flow cytometry and immunoblot. Similar transfection efficiency for each sample was assessed with the GFP expression plasmid pEGFP-C1 (BD Clontech, Heidelberg, Germany).

### Fluorescence-activated cell sorting analysis

Competitive binding to native EpCAM and binding to the above mentioned EpCAM glycosylation mutants was performed by flow cytometry using a fluorescence-activated cell sorting (FACS)Calibur device and the Cellquest pro software (Becton Dickinson, Heidelberg, Germany). Competitive binding assays were performed by preincubation of 2 × 10^5^ HCT-8 target cells with EpCAM specific mAbs C215 or VU-1D9 at the indicated amounts for 10 min. After a washing step with FACS buffer (PBS supplemented with 1% FCS and 0.1% sodium azide) cells were incubated with 2.5 *μ*g ml^−1^ FITC-labelled HO-3 mAb for 30 min, washed, and analysed by comparing the mean fluorescence intensity (MFI) relative to a mouse IgG2a, kappa isotype control (BD Pharmingen).

Analysis of HO-3 or C215 binding to native EpCAM mutants was done using HEK293 cells stably or transiently transfected with the above mentioned expression vectors. Therefore, 14–20 *μ*g ml^−1^ primary antibodies were each incubated with 2 × 10^5^ cells for 30 min. After washing three times, the PE or FITC-labelled rat-anti-mouse IgG detection antibody (Dianova, Hamburg, Germany) was added. Cells were washed again, and the MFI of the stained cells was determined.

### Epitope mapping

The EpCAM epitope recognised by the HO-3 antibody was defined by screening a peptide library covering amino acids 7–268. The 22-residue N-acetylated peptides with a 16 amino-acid overlap were directly synthesised on a cellulose membrane by spot synthesis as described by Reineke *et al* (1999). After incubation with HRP-labelled HO-3 antibody, the chemiluminescence signal intensity was quantified with an imaging system as BLU (Boehringer light units). The epitope mapping studies were carried out at Jerini Peptide Technologies (Berlin, Germany).

### Affinity measurement

The affinity of HO-3 and catumaxomab for the EpCAM protein was determined via surface plasmon resonance (SPR) using a Biacore 3000 device (Biacore AB, Uppsala, Sweden). The ECD of EpCAM was covalently coupled to a CM-5 sensor chip at low density (215 response units of EpCAM). Binding kinetics were performed with twofold serial dilutions of antibody at concentrations of 500–0.08 nM in running buffer (PBS, pH 7.4, 0.005% (v/v), polysorbate 20 – filtered and degassed) at 25°C and a flow rate of 25 *μ*l min^−1^. The regeneration procedure consisted of three injections of 10 *μ*l 2.5 M guanidinium hydrochloride followed by flushing of the sensor chip with running buffer for 5 min. Statistical and data processing were performed with BIA evaluation software 4.0.1 and GraphPad Prism 4.02 (GraphPad Software Inc., San Diego, CA, USA). All SPR experiments were carried out at Biaffin GmbH & Co KG (Kassel, Germany).

### Tetrazolium hydroxide cytotoxicity assay

Peripheral blood mononuclear cells (PBMC) (1 × 10^5^ cells) from healthy donors were isolated by ficoll density centrifugation and subsequently mixed with 1 × 10^4^ HCT-8 tumour cells in the presence of the indicated amounts of antibodies in 96-well flat bottomed plates (final volume 200 *μ*l RPMI-1640 medium containing 10% FCS, 1 mM sodium pyruvate, 2 mM L-glutamine, and 1 × non-essential amino acids). After 3 days of co-cultivation at 37°C and 5% CO_2,_ soluble PBMC were washed twice with PBS without Ca^2+^ Mg^2+^ (PAN Biotech, Aidenbach, Germany). Then, adherent tumour cells were stained with tetrazolium hydroxide (XTT) cell proliferation kit II (Roche Diagnostics GmbH, Mannheim, Germany) until the OD_650 nm-490 nm_ of the tumour cell control samples reached a value of about 1.5. Plates were measured with a VersaMax plate reader (Molecular Devices, CA, USA) with background values with medium alone subtracted. Killing efficiency of HO-3 and catumaxomab was assessed in a total of four independent experiments with fourfold determinations of each sample. Tumour cell killing (%) was calculated according to the formula: (OD_tumour cells_−OD_tumour cells+PBMC+antibody_)/(OD_tumour cells_–OD_PBMC_) × 100%.

## RESULTS

### C215, but not VU-1D9, competes with HO-3 for binding to EpCAM

**Figure 1:**
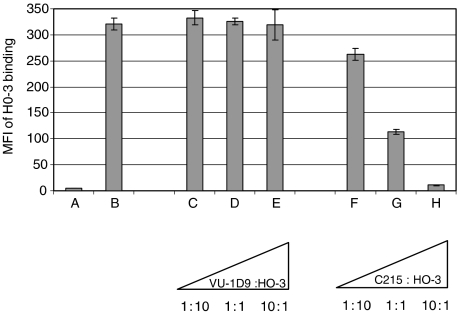
C215, but not VU-1D9, competes with mAb HO-3 for binding to wild-type EpCAM. Preincubation of HCT-8 tumour cells with mAb C215 blocked HO-3 binding by more than 50% relative to the positive control (B) with no competitor when the antibodies were added at equal concentrations (2.5 *μ*g ml^−1^). A 10-fold excess of mAb C215 (25 *μ*g ml^−1^) resulted in almost complete binding inhibition, which was down to the level of the isotype control (A; IgG2a, kappa). Preincubation of cells with VU-1D9 did not significantly influence HO-3 binding. Triangles indicate escalating amounts of competitor antibody with HO-3 concentration kept constant at 2.5 *μ*g ml^−1^. Each sample was measured three times; error bars indicate s.d. The experiment was repeated with similar results.

To confirm the specificity of the mAb HO-3 for EpCAM, we performed binding assays in the presence of the mAbs C215 and VU-1D9. Both C215 and VU-1D9 bind with high-affinity to the EGF-like domain I of EpCAM (Balzar *et al*, 1999). As depicted in Figure 1, C215 clearly inhibited the binding of HO-3 to EpCAM-positive HCT-8 cells in a dose-dependent manner, whereas VU-1D9 did not. A 10-fold excess of preadded mAb C215 entirely blocked HO-3 binding to EpCAM-positive cells suggesting that both antibodies recognise similar epitopes.

### HO-3 binds within the EGF I repeat of EpCAM

**Figure 2:**
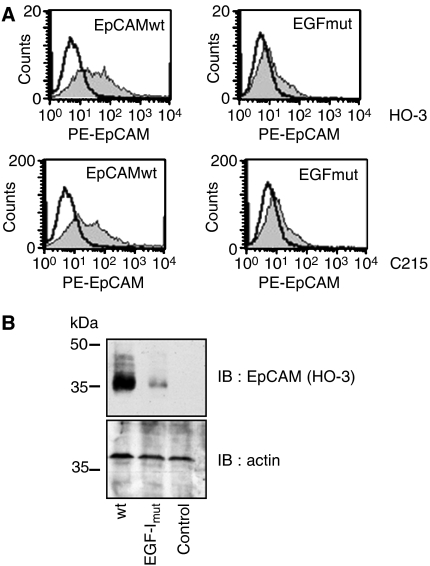
HO-3 binds within EGF-like domain I of EpCAM. (**A**) HEK293 were transiently transfected with wild-type EpCAM or the EpCAM EGF-I mutant and stained with HO-3 (grey curves; upper panels) or C215 (grey curves; lower panels) in combination with a PE-conjugated secondary antibody. As a control, primary Ab was omitted (black line). (**B**) Transfections were performed as in (**A**), proteins were separated by 10% SDS–PAGE, and EpCAM was visualised with the mAb HO-3. For a control, levels of actin were assessed on the same membrane. Shown are the representative results from three independent experiments.

The C215 antibody binds within the EGF-like domain I of EpCAM (Bjork *et al*, 1993). Given the fact that C215, but not VU-1D9, competed for the binding of HO-3 to EpCAM, we anticipated similar binding sites for C215 and HO-3. To test our assumption, flow cytometry assays were conducted to assess the binding activity of HO-3 to an EpCAM deletion mutant lacking the EGF-like domain I (EGFmut; amino acids 27–59). Indeed, the reactivity of HO-3 towards the deletion mutant was strongly impaired when compared to the recognition of an EpCAM wild-type control (Figure 2A). A similar loss of binding to the EGF-like domain mutant was observed for the mAb C215, which was used as a control. However, in both cases residual binding activity could be detected. A comparably strong, though not complete, loss of binding activity for mAbs HO-3 and C215 was observed when EpCAM-EGFmut was detected in its denatured form by immunoblotting (Figure 2B). These results define the binding specificity of HO-3 and C215 within EpCAM's EGF-like domain I, but point towards additional binding sites beyond this particular protein stretch.

### HO-3 recognises a discontinuous epitope, having three binding sites in EpCAM

Next, the binding specificity of HO-3 was fine-mapped by screening a peptide library covering the complete ECD of EpCAM (amino acids 7–268). We analysed 22-residue peptides, with a 16-amino-acid overlap, to achieve high-resolution mapping. The synthetic peptide library included the amino acid sequence ranging from residues 31 to 52 of wild-type EpCAM that was most strongly targeted by mAb C215 (Bjork *et al*, 1993). Screening the peptide library with the C215 control antibody confirmed the recognition of two peptides at amino acid positions 31–52 and 103–124 (Table 2). Interestingly, mAb HO-3 recognised a conformational epitope, indicating the presence of three EpCAM binding sites. A comparison of the amino acid sequences recognised by C215 and HO-3 revealed an overlap in the epitope within the EGF-like domain I. This common binding region readily explains the competitive reactivity of both investigated antibodies. However, an additional major HO-3 binding site was localised within the EGF-like domain II between amino acids 67 and 88. A third binding site situated more C-terminally (103–124) could also be identified. Notably, there was no cross-reaction with murine EpCAM (data not shown).

### HO-3 and the corresponding trAb catumaxomab bind with high affinity to EpCAM

Binding of HO-3 to EpCAM-positive target cells, as measured by flow cytometry, pointed toward a high-affinity antibody (data not shown). Surface plasmon resonance was applied to determine the exact binding constants for HO-3. For this purpose, the recombinant ECD of native EpCAM was expressed in yeast, and the antibody–antigen interaction was investigated with Biacore technology. Both HO-3 and the corresponding trAb catumaxomab revealed high affinities, with low dissociation constants of 5.5 × 10^−10^ and 5.6 × 10^−10^ M, respectively (Table 3). Owing to the low EpCAM antigen density covalently linked to the sensor chip, these values mainly reflect monovalent binding. After increasing EpCAM density avidity effects of bivalent HO-3 binding, indicated by reduced-off rates, were observed (data not shown).

### Recognition of EpCAM by HO-3 is independent of EpCAM's glycosylation status

The amino acid sequence of EpCAM comprises three putative N-glycosylation sites (NGT/S) (Chong and Speicher, 2001). Differential usage of these N-glycosylation sequences results in the generation of EpCAM variants of different sizes ranging from 37 to 42 kDa. Notably, a hyperglycosylation pattern was observed in head and neck carcinoma when compared with healthy EpCAM-positive epithelium (Pauli *et al*, 2003).

EpCAM was strongly glycosylated in 77% of cases of head and neck carcinomas studied (*n*=44). When comparing pairs of carcinomas and EpCAM-positive thyroid tissue from the same patients (*n*=26), a significant hyperglycosylation of EpCAM in tumours was observed in 80.7% of specimen. Hence, we wondered whether protein glycosylation might eventually play a role in antibody recognition, as this would have serious repercussions on therapeutic application.

**Figure 3:**
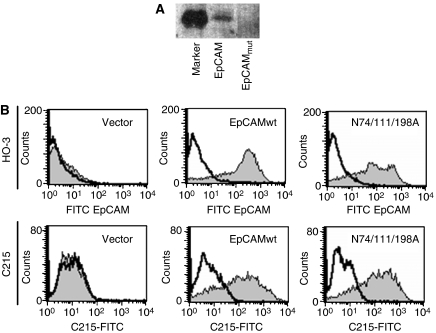
HO-3 and C215 recognise EpCAM independently of the glycosylation status. (**A**) HEK293 cells were stably transfected with an expression vector for wild-type EpCAM and a triple mutant lacking all glycosylation sites (EpCAM_mut_). Equal amounts of cell lysates of each cell lines were separated by 10% SDS–PAGE and subjected to glycostaining. Staining was assessed with the FLA 5000 scanning device (Fuji). M represents an internal marker provided by the manufacturers. (**B**) HEK293 cells stably expressing wild-type EpCAM, a triple mutant lacking all *N*-glycosylation consensus sites (N74/111/198A; EpCAM_mut_), or the empty vector for a control were stained with HO-3 (grey curves; upper panels) or C215 (grey curves; lower panels) in combination with a FITC-conjugated secondary antibody. As a control, primary antibody was omitted (black line). All data are representative results from three independent experiments.

Point mutations were sequentially inserted into the extracellular domain of wild-type EpCAM to exchange asparagine residues in each N-glycosylation consensus site to an alanine (*N* → *A*) to generate a triple mutant (N74/111/198A). This EpCAM triple mutant lacking all consensus asparagine residues (N74A, N111A, N198A) was unglycosylated, as shown on glycostaining (Figure 3A). Thereafter, independent bulk cultures of EpCAMwt and N74/111/198A stable HEK293 transfectant were stained with mAb HO-3 or C215 and measured in a flow cytometer. EpCAM was detected by both the HO-3 and C215 antibodies and independently of the glycosylation status, that is EpCAMwt and N74/111/198A were equally well bound (Figure 3B). Hence, binding of HO-3 to EpCAM is not affected by the glycosylation status of the protein.

### Catumaxomab, but not HO-3, efficiently mediates tumour cell killing *in vitro*

**Figure 4:**
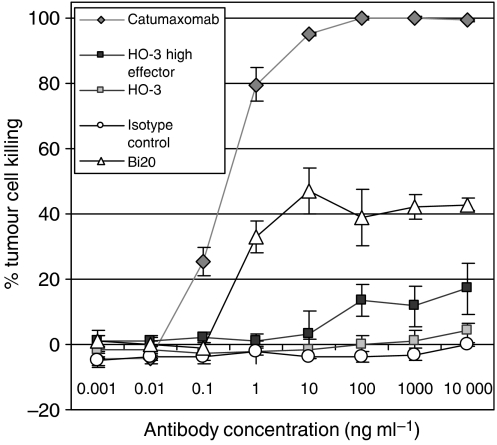
TrAb catumaxomab is 1000-fold more potent than HO-3 in eliciting PBMC-mediated killing of EpCAM-positive carcinoma cells. Comparison of trAb and mAb mediated cytotoxicity against EpCAM-positive tumour cells *in vitro*. HCT-8 tumour cells and PBMCs were co-cultured at ratios of 10 : 1 in the presence of trAbs catumaxomab (anti-EpCAM × anti-CD3), Bi20 (anti-CD20 × anti-CD3) or mAb HO-3 at the indicated concentrations. Additional approaches with high effector ratios of 50 : 1 were performed for H0-3 and mouse IgG2a isotype control MmT1 (anti-mouse Thy-1.2). After 3 days, tumour cell killing was measured by XTT staining. Alloreactivity of PBMC without antibody was not significant (0–2%). Data points display mean values of four determinations with s.d. Data are representative results from four independent experiments using PBMCs from different donors.

The capacity of HO-3 or the therapeutic trAb catumaxomab to induce the killing of EpCAM-positive tumour cells by immune effector cells was compared *in vitro*. The cytotoxic potential of PBMC was assessed by XTT staining after co-cultivation of PBMC and EpCAM-positive tumour targets for 3 days. Significant tumour cell eradication was observed after addition of trAb catumaxomab at low concentration of 0.1 ng ml^−1^. Tumour cells were quantitatively killed at concentrations greater than 10 ng ml^−1^ (Figure 4). EpCAM-independent killing induced by CD20-specific trifunctional control antibody Bi20 was approximately 40%. Similar results were obtained using PBMC from different donors (*n*=4). In sharp contrast, HO-3 mediated a weak killing efficiency only at a favourable effector to target ratio of 50 : 1 and at concentrations as high as 100 ng ml^−1^. As expected, HO-3-mediated killing was specific since mouse IgG2a isotype control antibody did not display any anti-tumoral activity. In summary, catumaxomab and HO-3 exhibit a strong potency difference with regard to their ability to induce the killing of EpCAM-positive cancer cells *in vitro*.

## DISCUSSION

Soon after its discovery, the high frequency of EpCAM overexpression in many epithelial tumours suggested it as an attractive target for antibody-based therapies (Herlyn *et al*, 1979; Gottlinger *et al*, 1986a; Riethmuller *et al*, 1994). Since then, there have been a plethora of therapeutic approaches guided by the idea of targeting tumour cells via EpCAM. These strategies include the use of murine and humanised monoclonal antibodies, retroviral constructs, vaccination protocols, as well as bispecific and trifunctional antibodies (Balzar *et al*, 1999; Armstrong and Eck, 2003; Kufer *et al*, 2004; Baeuerle and Gires, 2007).

Here, we characterise the new mAb HO-3 that constitutes the EpCAM-binding arm of the corresponding therapeutic trAb catumaxomab. The binding features of this mouse IgG2a antibody are described in detail. Studies with EpCAM deletion mutants and binding assays in the presence of the competing mAb C215 pointed toward a recognition site within the EGF-like domain I in the extracellular domain of EpCAM. This was confirmed by fine-mapping the antigenic epitope through the screening of an overlapping peptide library which revealed a discontinuous epitope with two major binding sites within the EGF I and the EGF II domains, respectively. A third minor binding site located C-terminally to EGF II was also identified. Thus, HO-3, just like the great majority of EpCAM-specific antibodies, targets EpCAM mainly within the EGF like domains, which represent the most immunogenic components of this protein (Balzar *et al*, 1999). Driven by the knowledge of differential glycosylation of EpCAM in normal *vs* carcinoma tissue (Pauli *et al*, 2003), we assessed the dependence of HO-3 binding on the glycosylation status of the molecule. A potential impact of post-translational modification of EpCAM on the binding of therapeutic antibodies available so far remained entirely unexplored. Nevertheless, this may be of major importance as suggested by the tumour antigen MUC-1 whose hypoglycosylated variant expressed in tumour tissue is differentially recognised by mucin-specific antibodies (Grinstead *et al*, 2002; Karsten *et al*, 2005; Li and Cozzi, 2007). However, HO-3 binding to EpCAM was entirely independent of the presence of glycosyl residues on the target protein. Of note, the glycosylation grade of EpCAM derived from head and neck squamous cell carcinomas varied, and it is as yet unclear whether weakly or non-glycosylated EpCAM variants may appear on other tumours. Taking this into account, mAbs such as HO-3 that are insensitive to the glycosylation status of EpCAM have an expanded potency to target a great variety of EpCAM-positive tumours.

Certainly, the most interesting aspect of new antibodies against EpCAM is their therapeutic value. Upto now, edrecolomab (Panorex®) is one of the best characterised EpCAM-specific therapeutic antibodies (Gottlinger *et al*, 1986b; Goodwin *et al*, 1987). However, the clinical benefit of this mouse IgG2a mAb is limited. Having first shown impressive reduction in mortality in Duke's C colorectal cancer patients compared with the control arm consisting of surgery alone, edrecolomab proved to be less effective than chemotherapy in a proximate phase III study (Riethmuller *et al*, 1994; Punt *et al*, 2002). The low affinity of the antibody is one critically discussed aspect. Improved treatment results with the strongly binding mAb 323/A3 in a mouse xenograft tumour model speak in favour of the clinical development of high-affinity anti-EpCAM therapeutic antibodies (Velders *et al*, 1995). However, the maximum tolerated dose of the high-affinity EpCAM-targeting mAb ING-1, as determined in a dose escalation study, turned out to be low, with only 0.1 mg kg^−1^ *i.v.* Additionally, severe toxicity in the form of acute pancreatitis occurred at higher concentrations (de Bono *et al*, 2004). Keeping the affinity low but improving ADCC effector function by chimaerisation or generation of fully human IgG1 antibodies may be a more promising method. With adecatumumab (MT201), such a novel human IgG1 anti-EpCAM antibody is presently in clinical development (Naundorf *et al*, 2002). However, common mAb effector functions such as ADCC and CDC may not be strong enough in terms of cancer therapy. High antibody dosages and continued administration are required to compensate for the endogenous serum IgG that competes for binding to Fcγ receptors on immune effector cells, and complement inhibitors that are expressed on tumour cells (Gorter and Meri, 1999; Preithner *et al*, 2006).

TrAbs represent a novel and promising alternative to improve anti-cancer efficacy. Owing to the simultaneous recruitment and activation of T cells and accessory immune cells, a variety of effector functions, such as ADCC, phagocytosis, and T-cell mediated cytotoxicity, are induced in a combinatorial fashion (Zeidler *et al*, 1999, 2000; Riesenberg *et al*, 2001). This may lead to a 1000-fold increase in anti-tumour potency as was demonstrated in the present study. The trAb catumaxomab (removab®), which is based on the mAb HO-3 and displays specificities for EpCAM and CD3, is far more potent in inducing tumour cell killing by PBMC than is HO-3 alone. Besides the trAb-mediated concerted attack by different types of effector cells, the high affinity of the anti-EpCAM binding arm may contribute to its effectiveness as an anti-tumour agent. Not only for HO-3, but also for monovalently binding catumaxomab, we measured a remarkably low dissociation constant of 5.6 × 10^−10^ M. As EpCAM is not tumour-specific, but rather is tumour-associated and also expressed on normal epithelial tissue (Balzar *et al*, 1999), high affinity conventional mAbs may be disadvantageous, with therapeutic concentrations that cause severe adverse events. This is different with trAbs, which are administered in the clinic at very low dosages in the microgram range. However, to guarantee effective and stable opsonisation of tumour cells, a very high-affinity and a slow-off rate of the therapeutic antibody is required, especially when administered at non-saturating concentrations. Hence, we propose that the high-affinity and the assessed off rate of only 3.3±0.3 × 10^−5^ s^−1^ of the anti-EpCAM targeting arm is one of the crucial features of catumaxomab.

The therapeutic effectiveness of this novel trAb has not only been demonstrated *in vitro* and in different *in vivo* tumour models (Lindhofer *et al*, 1996; Ruf and Lindhofer, 2001; Schmitt *et al*, 2004). Catumaxomab's clinical benefit was recently verified when it was used to treat patients suffering from malignant ascites (Heiss *et al*, 2005). In this prospective study, catumaxomab was applied intraperitoneally, was well tolerated, and effectively diminished the local tumour cell burden and ascites fluid accumulation.

Biodistribution studies in EpCAM transgenic mice suggested preferential access of EpCAM-specific mAbs to tumour cells in spite of a background expression of EpCAM on healthy tissue (McLaughlin *et al*, 2001). This demonstrates the suitability of using EpCAM for antibody-based cancer therapy, in principle. In conclusion, the application of high-affinity and effective trAbs administered locally at very low concentrations may re-open the therapeutic window for immunotherapy of EpCAM expressing tumours.

## Figures and Tables

**Table 1:** Primers used for PCR-based site directed mutagenesis of epithelial cell adhesion molecule (EpCAM)

*Primers used for amplification of PCR products after mutagenesis*
External primer *1*: 5′-GGCACCAAAATCAACGGGAC-3′
External primer *2*: 5′-AGGGTCAAGGAAGGCACGG-3′

*Primers used for generation of glycosylation mutants*
*N74A-*fw int. primer (internal primer 1)
5′-TGGTGATGAAGGCAGAAATG**GCA**GGCTCAAAACTTGGGAGAAG-3′
*N74A-*bw int. primer (internal primer 3)
5′-CTTCTCCCAAGTTTTGAGCC**TGC**CATTTCTGCCTTCATCACCA-3′
*N111A-*fw int. primer (internal primer 2)
5′-AGGCCAAGCAGTGC**GCG**GGCACCTCCACGTG-3′
*N111A-*bw int. primer (internal primer 4)
5′-CACGTGGAGGTGCC**CGC**GCACTGCTTGGCCT-3′
*N198-*fw int. primer
5′-AATGTTATCACTATTGATCTGGTTCAA**GCC**TCTTTCTCAAAAAACTCAGAATGATGTG-3′
*N198-*bw int. primer
5′-CACATCATTCTGAGTTTTTTGAGAAGA**GGC**TTGAACCAGATCAATAGTGATAACATT-3′

*Primers used for generation of EGF I deletion mutant*
Internal primer 1
5′-CACCAAACATTTGGCAGCCAGCTTTGAACATTCTTCCTGAGCTGCGGCAAA-3′
Internal primer 2
5′-TTTGCCGCAGCTCAGGAAGAATGTTCAAAGCTGGCTGCCAAATGTTTGGTG-3′

Abbreviations: A=alanine; bw=reverse; fwd=forward; int=internal; N=asparagines.

**Table 2:** Identification of EpCAM peptides recognised by HO-3 and C215.

**AA position**	**Peptide sequence**	**Signal intensity (BLU)**	**mAb**
49–70	TSVGAQNTVICSKLAAKCLVMK	6829	HO-3
67–88	LVMKAEMNGSKLGRRAKPEGAL	18813	HO-3
175–196	QLDPKFITSILYENNVITIDLV	3543	HO-3
31–52	NYKLAVNCFVNNNRQCQCTSVG	16703	C215
103–124	GLFKAKQCNGTSTCWCVNTAGV	12006	C215

Screening a peptide library covering the complete ECD of EpCAM showed that the listed peptides had the strongest interaction with the indicated antibody. Signals ranging from 30 to 800 Boehringer Units (BLU) were considered to be non-specific (background).

**Table 3:** Determination of kinetic constants for antibody-EpCAM interactions.

**Antibody**	**k_ass_ (M^−1^s^−1^)**	**k_diss_ (s^−1^)**	**K_D_ (M)**
HO-3	5.4±1.6 × 10^4^	2.7±0.8 × 10^−5^	5.5 × 10^−10^±0.19
Catumaxomab	6.1±1.5 × 10^4^	3.3±0.3 × 10^−5^	5.6 × 10^−10^±0.12

Interaction between the ECD of native EpCAM and mAb was measured by surface plasmon resonance. Both the parental mAb HO-3 and the trAb variant catumaxomab displayed high-affinity binding with slow off rates.
